# Supplementary material for: Oncolytic virus with an immune stimulatory payload for osteosarcoma therapy
Source: Cancer Immunol Immunother. 2026 Jun 20;75(10):222. doi: 10.1007/s00262-026-04458-0 (PMC13578186; doi:10.1007/s00262-026-04458-0)
Supplement: Supplementary file 1 [file 262_2026_4458_MOESM1_ESM.pdf]

Figure S1.

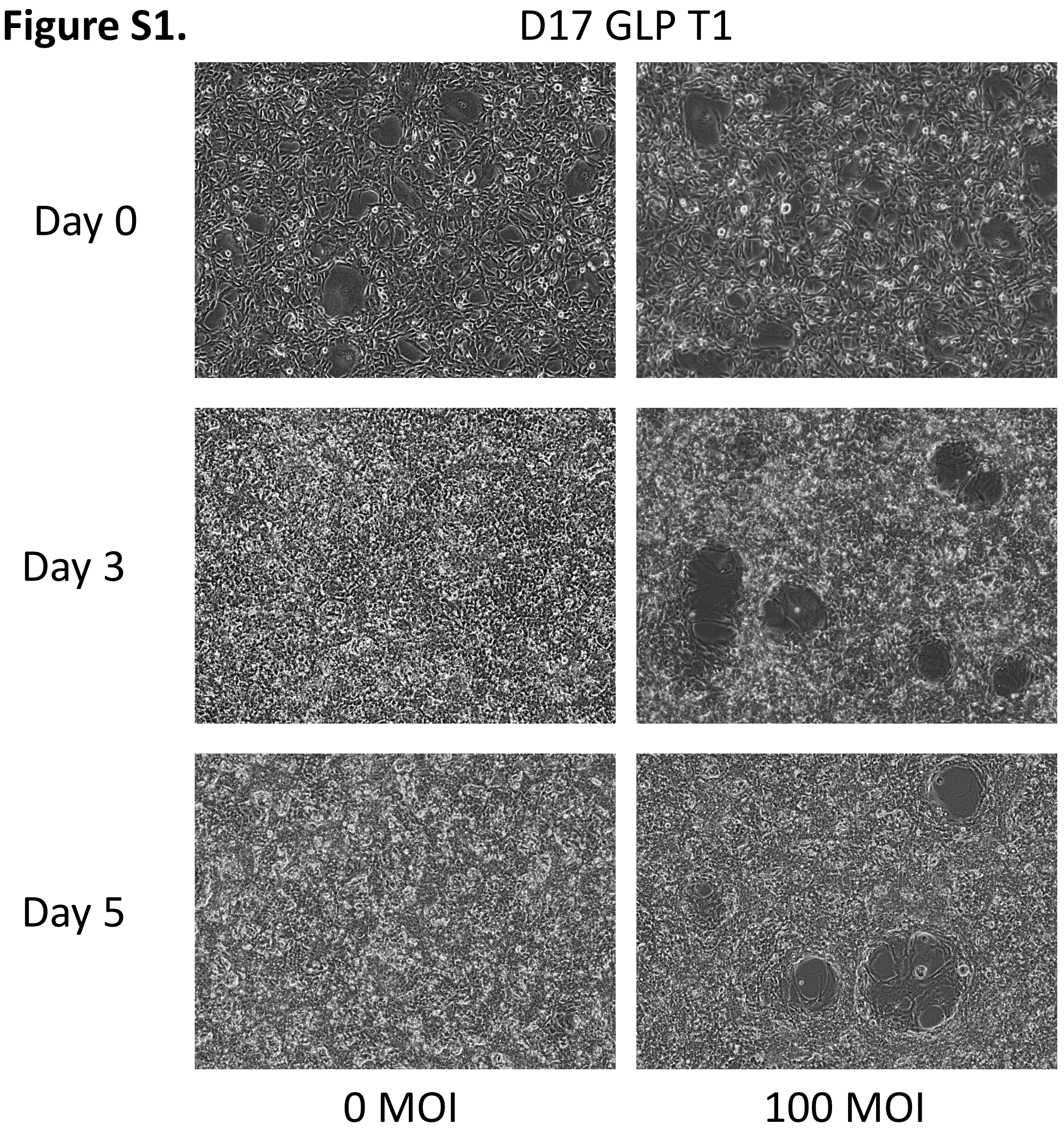

D17 GLP cells were infected with CAV-AU-M3 at MOI 0 and 100, and 1000, and images were visualized on day 0, 3, and 5 post-infection by inverted microscopy (Keyence) at 10X magnification. This represents three independent experiments.

Figure S2.

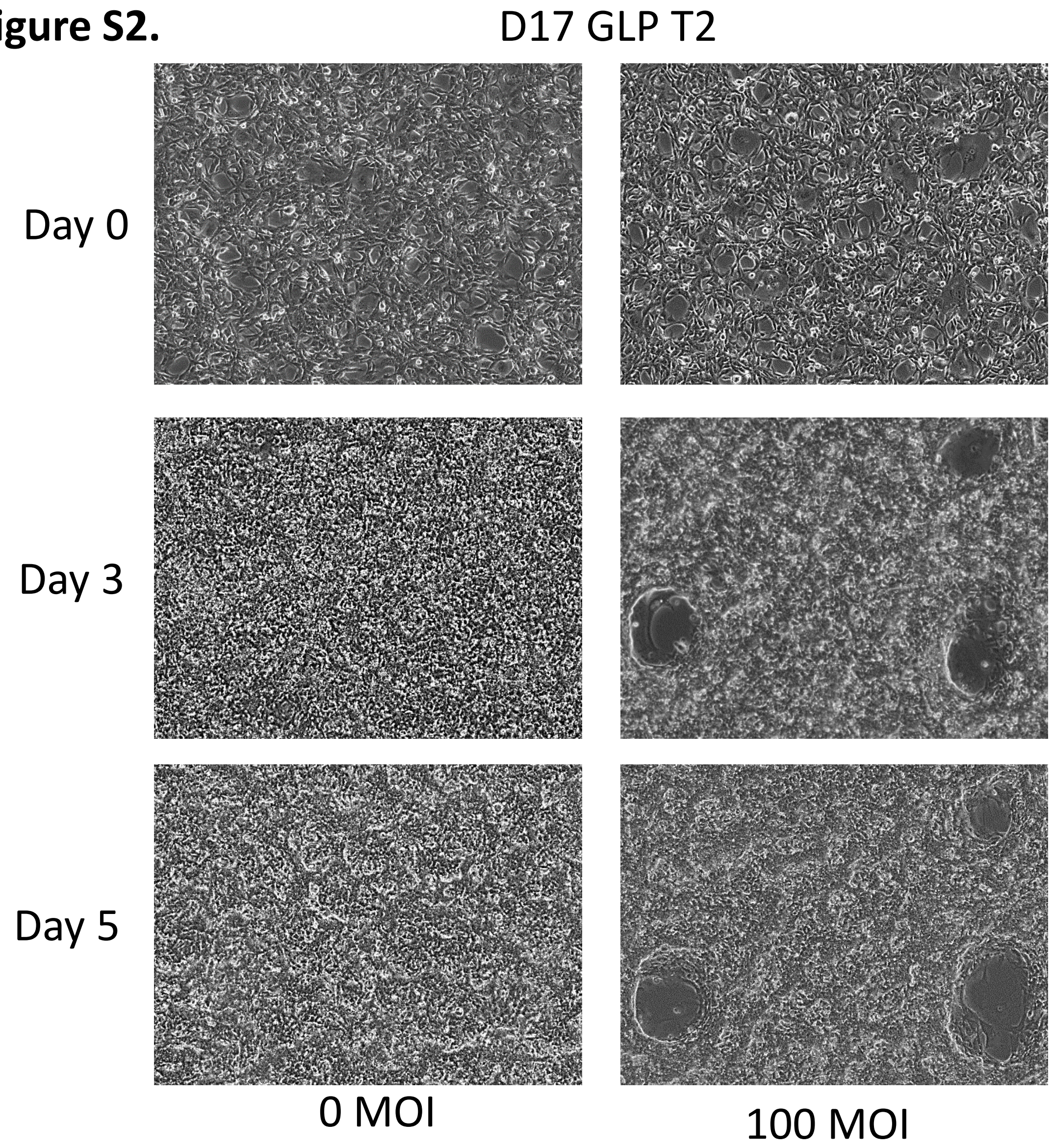

D17 GLP cells were infected with CAV-AU-M3 at MOI 0 and 100, and 1000, and images were visualized on day 0, 3, and 5 post-infection by inverted microscopy (Keyence) at 10X magnification. This represents three independent experiments.

Figure S3.

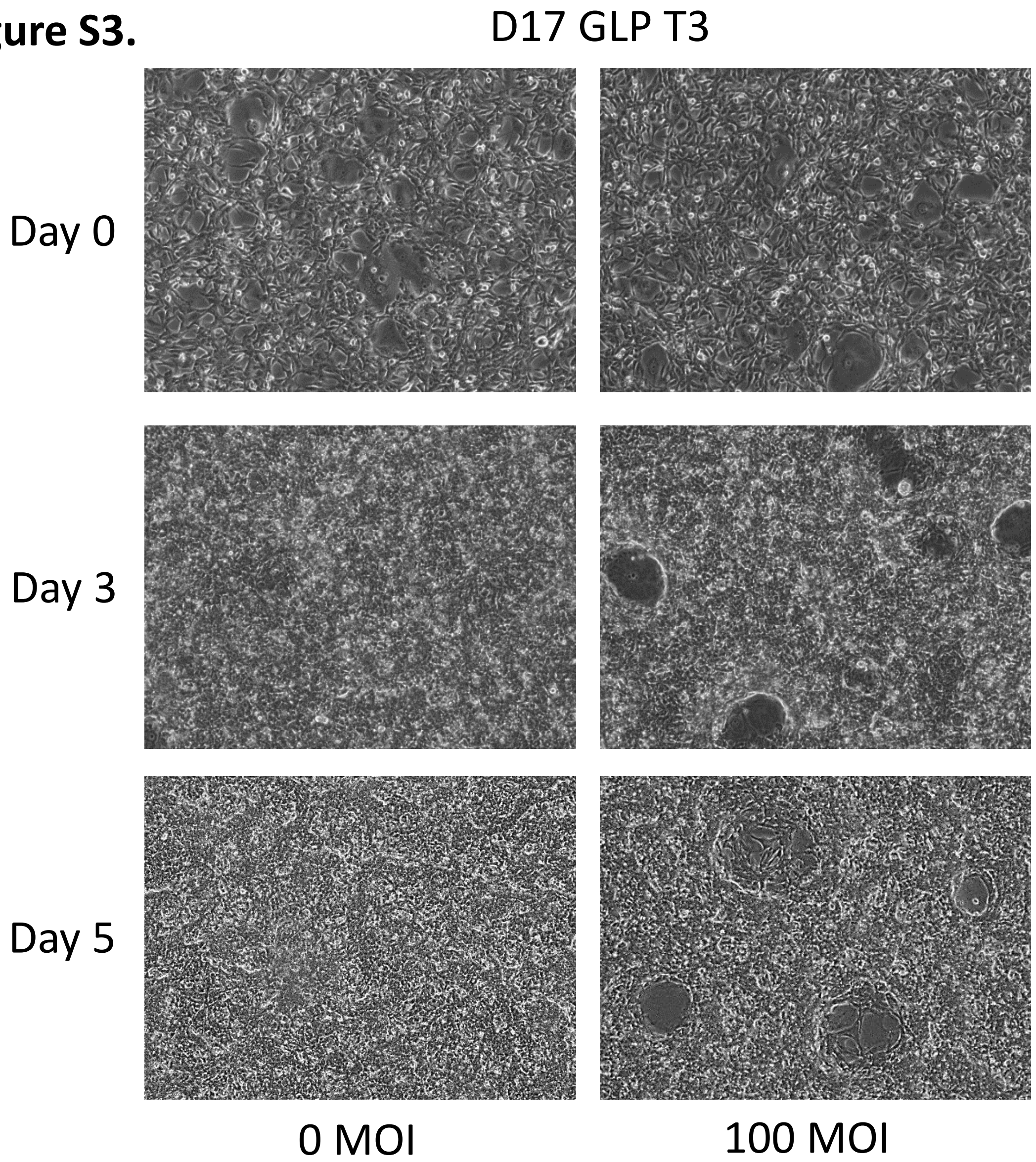

D17 GLP cells were infected with CAV-AU-M3 at MOI 0 and 100, and 1000, and images were visualized on day 0, 3, and 5 post-infection by inverted microscopy (Keyence) at 10X magnification. This represents three independent experiments.

Figure S4.

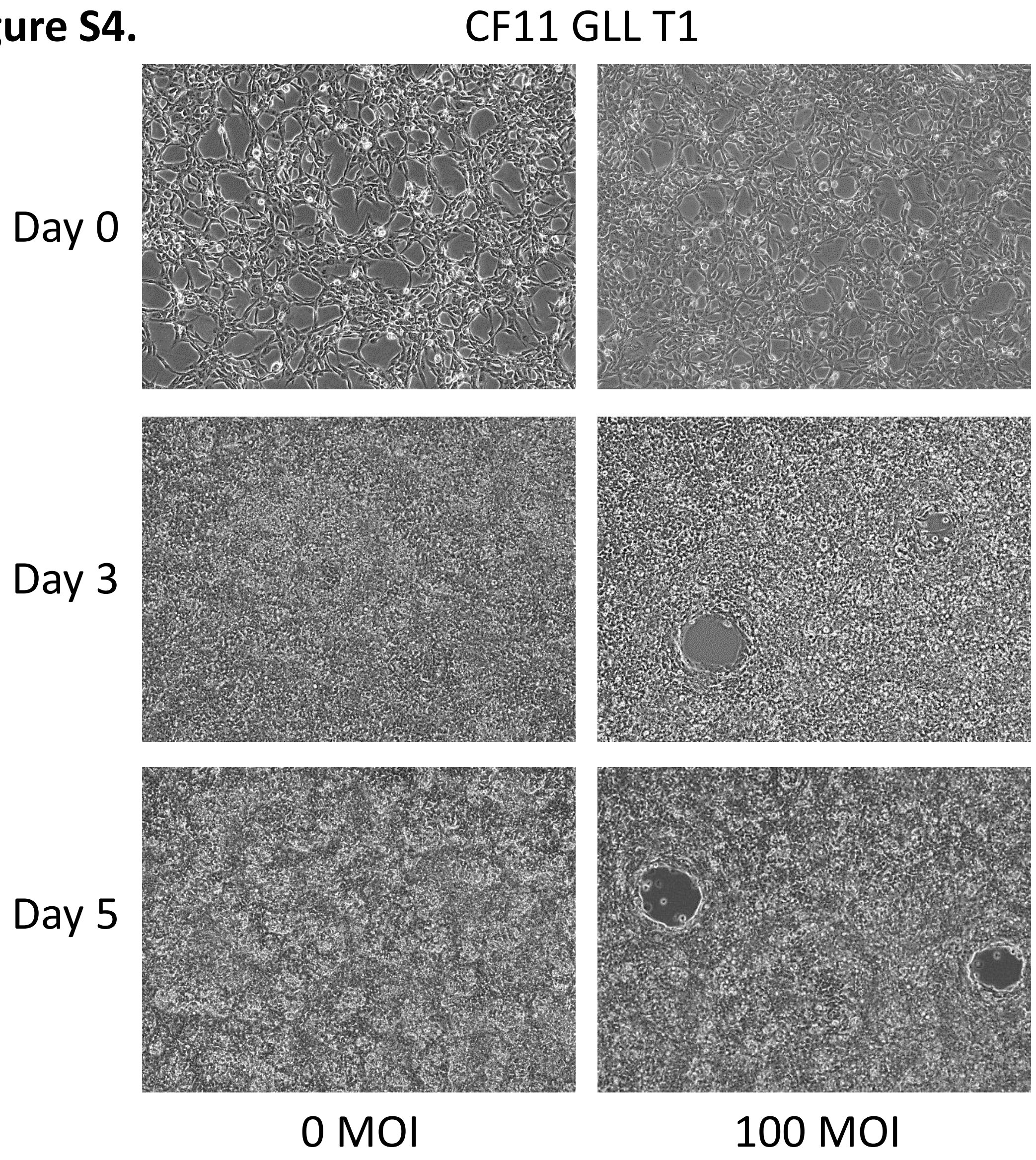

CF11 GLL cells were infected with CAV-AU-M3 at MOI 0 and 100, and 1000, and images were visualized on day 0, 3, and 5 post-infection by inverted microscopy (Keyence) at 10X magnification. This represents three independent experiments.

Figure S5.

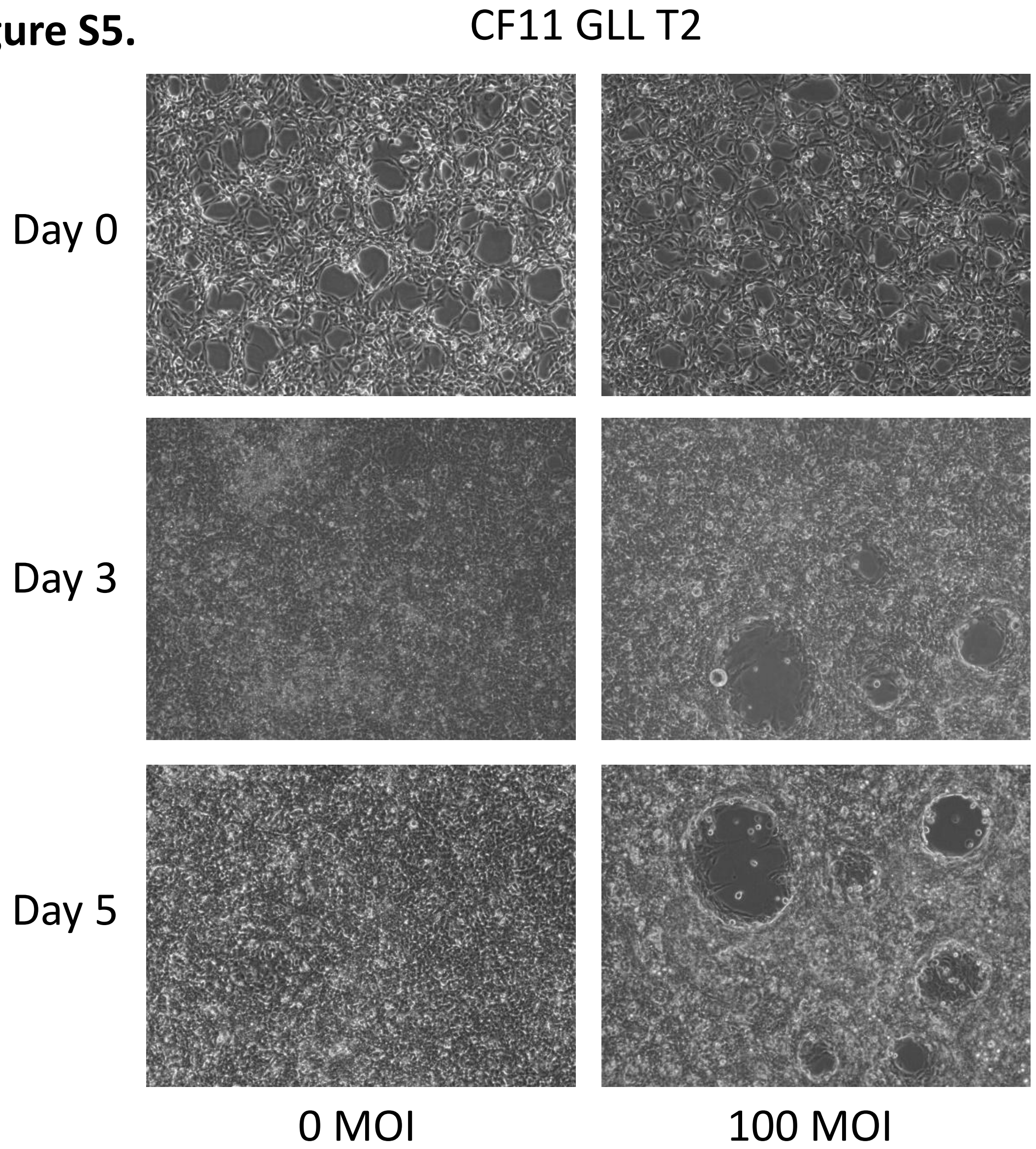

CF11 GLL cells were infected with CAV-AU-M3 at MOI 0 and 100, and 1000, and images were visualized on day 0, 3, and 5 post-infection by inverted microscopy (Keyence) at 10X magnification. This represents three independent experiments.

Figure S6.

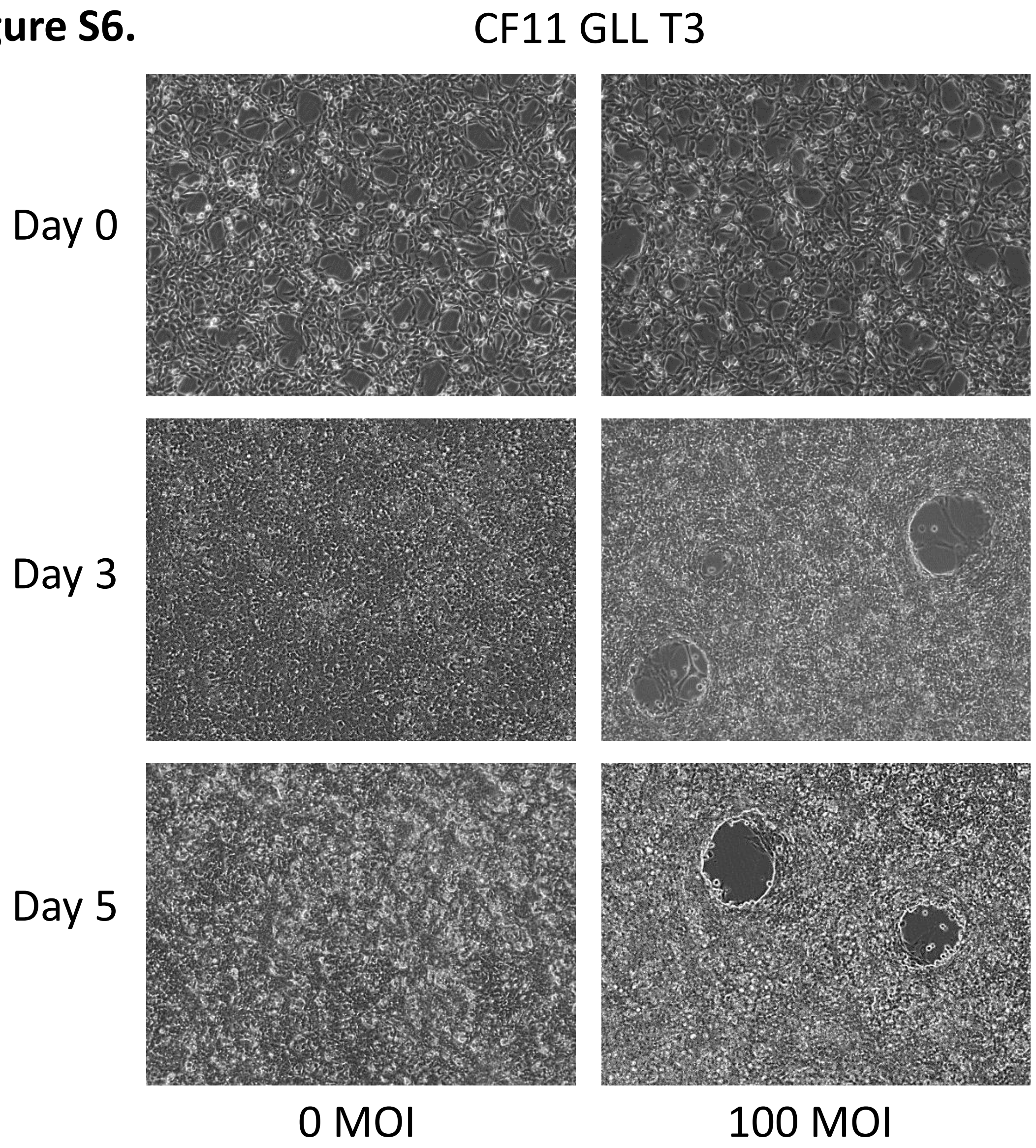

CF11 GLL cells were infected with CAV-AU-M3 at MOI 0 and 100, and 1000, and images were visualized on day 0, 3, and 5 post-infection by inverted microscopy (Keyence) at 10X magnification. This represents three independent experiments.

Figure S7.

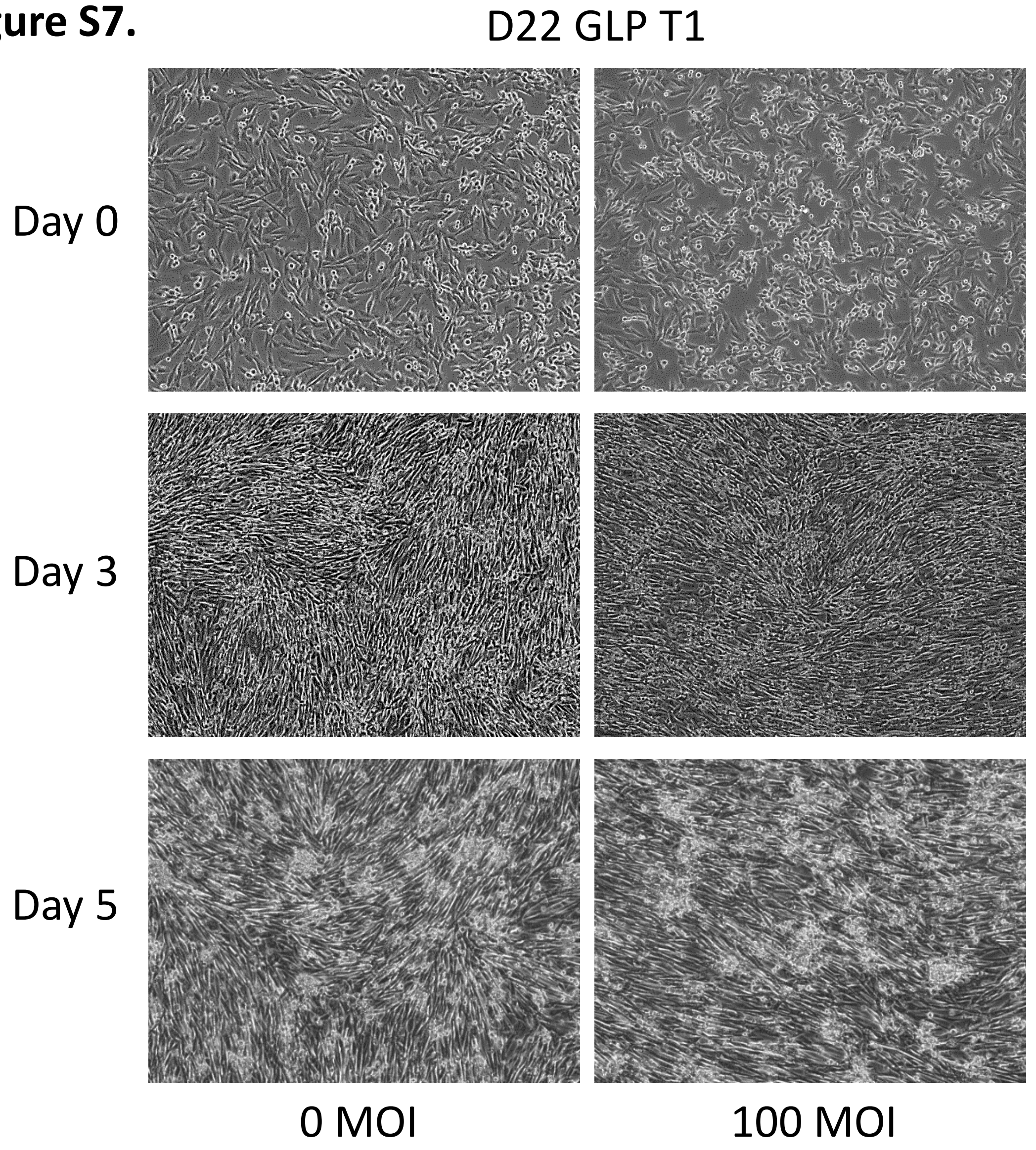

D22 GLP cells were infected with CAV-AU-M3 at MOI 0 and 100, and 1000, and images were visualized on day 0, 3, and 5 post-infection by inverted microscopy (Keyence) at 10X magnification. This represents three independent experiments.

Figure S8.

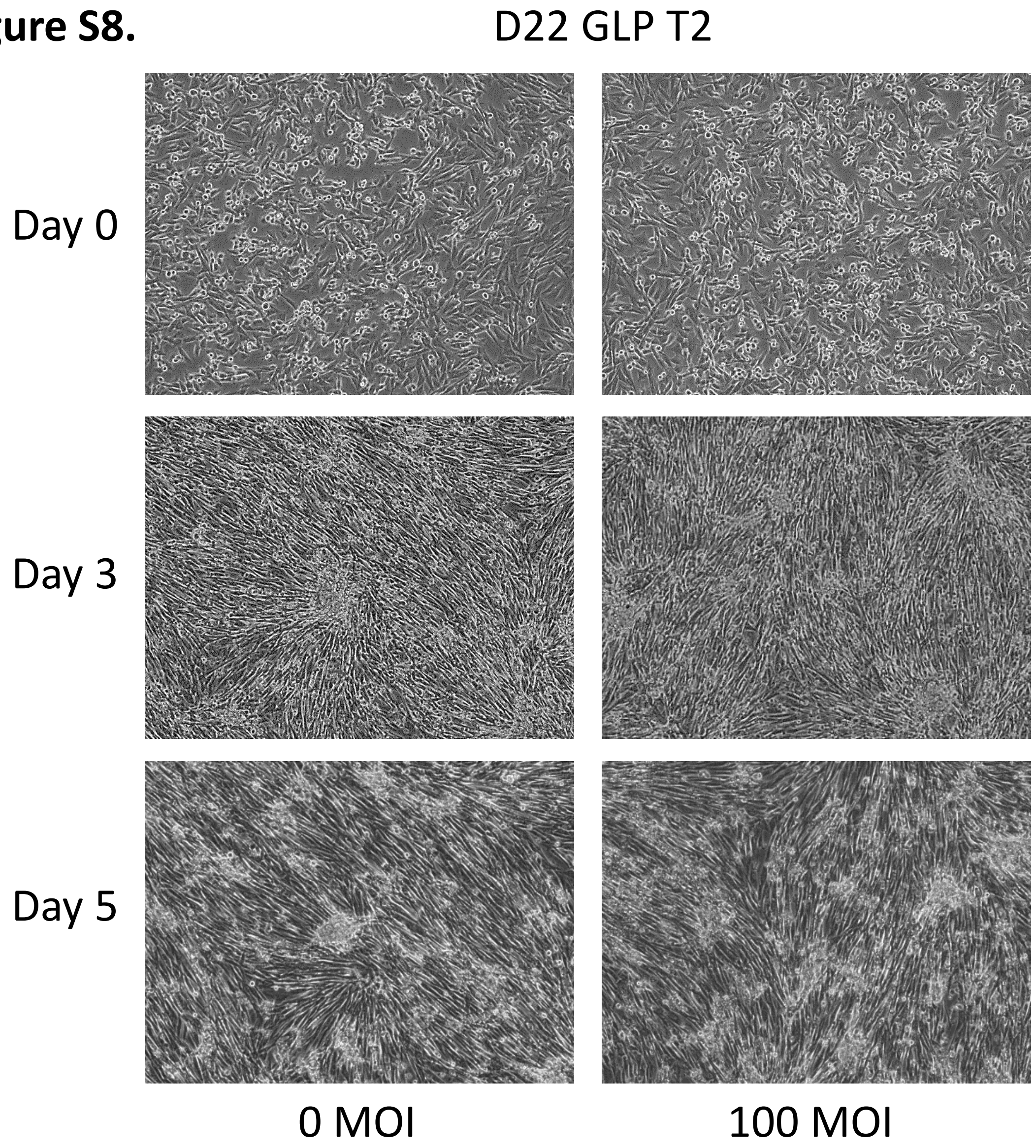

D22 GLP cells were infected with CAV-AU-M3 at MOI 0 and 100, and 1000, and images were visualized on day 0, 3, and 5 post-infection by inverted microscopy (Keyence) at 10X magnification. This represents three independent experiments.

Figure S9.

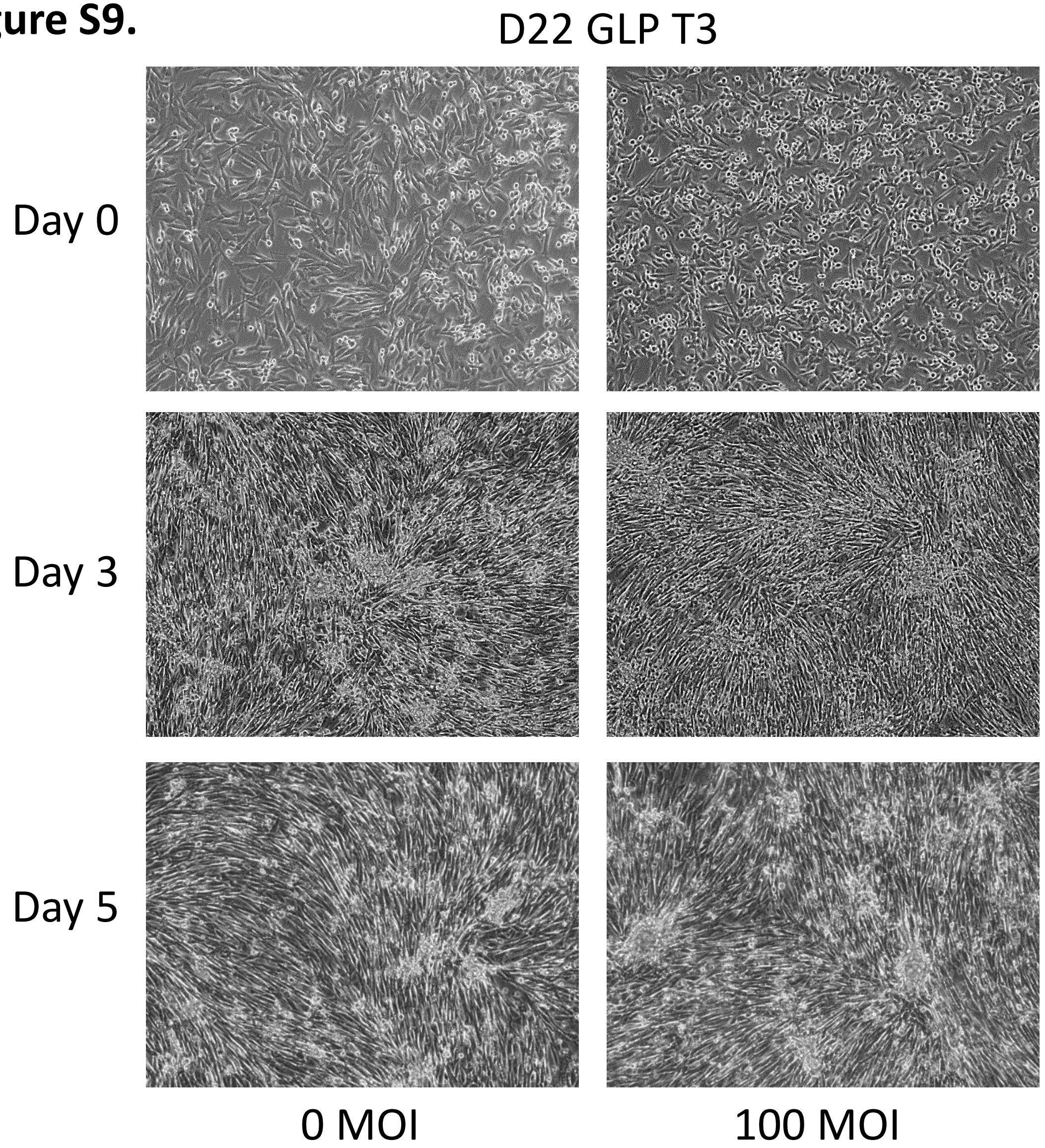

D22 GLP cells were infected with CAV-AU-M3 at MOI 0 and 100, and 1000, and images were visualized on day 0, 3, and 5 post-infection by inverted microscopy (Keyence) at 10X magnification. This represents three independent experiments.

Figure S10.

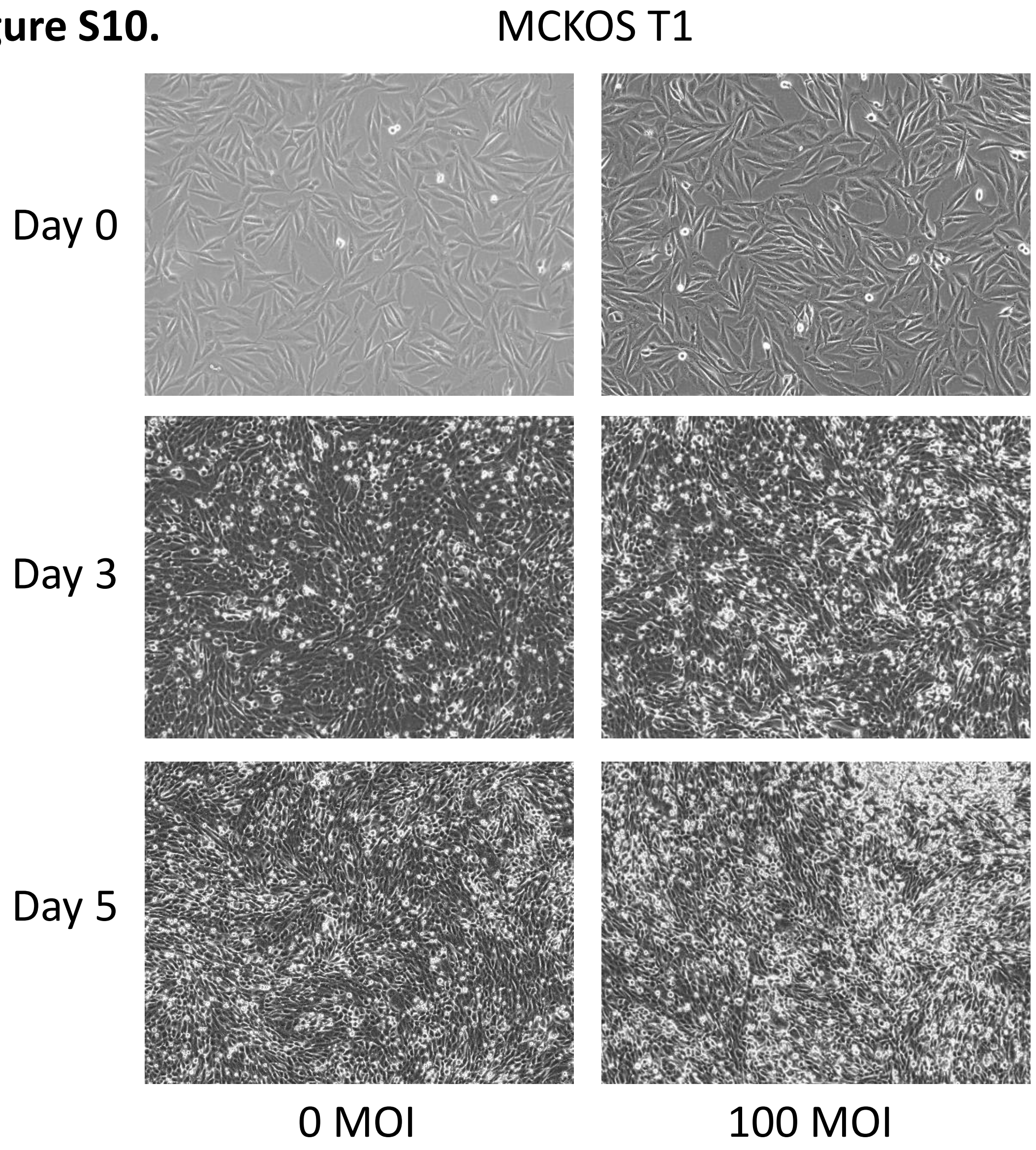

MCKOS cells were infected with CAV-AU-M3 at MOI 0 and 100, and 1000, and images were visualized on day 0, 3, and 5 post-infection by inverted microscopy (Keyence) at 10X magnification. This represents three independent experiments.

Figure S11.

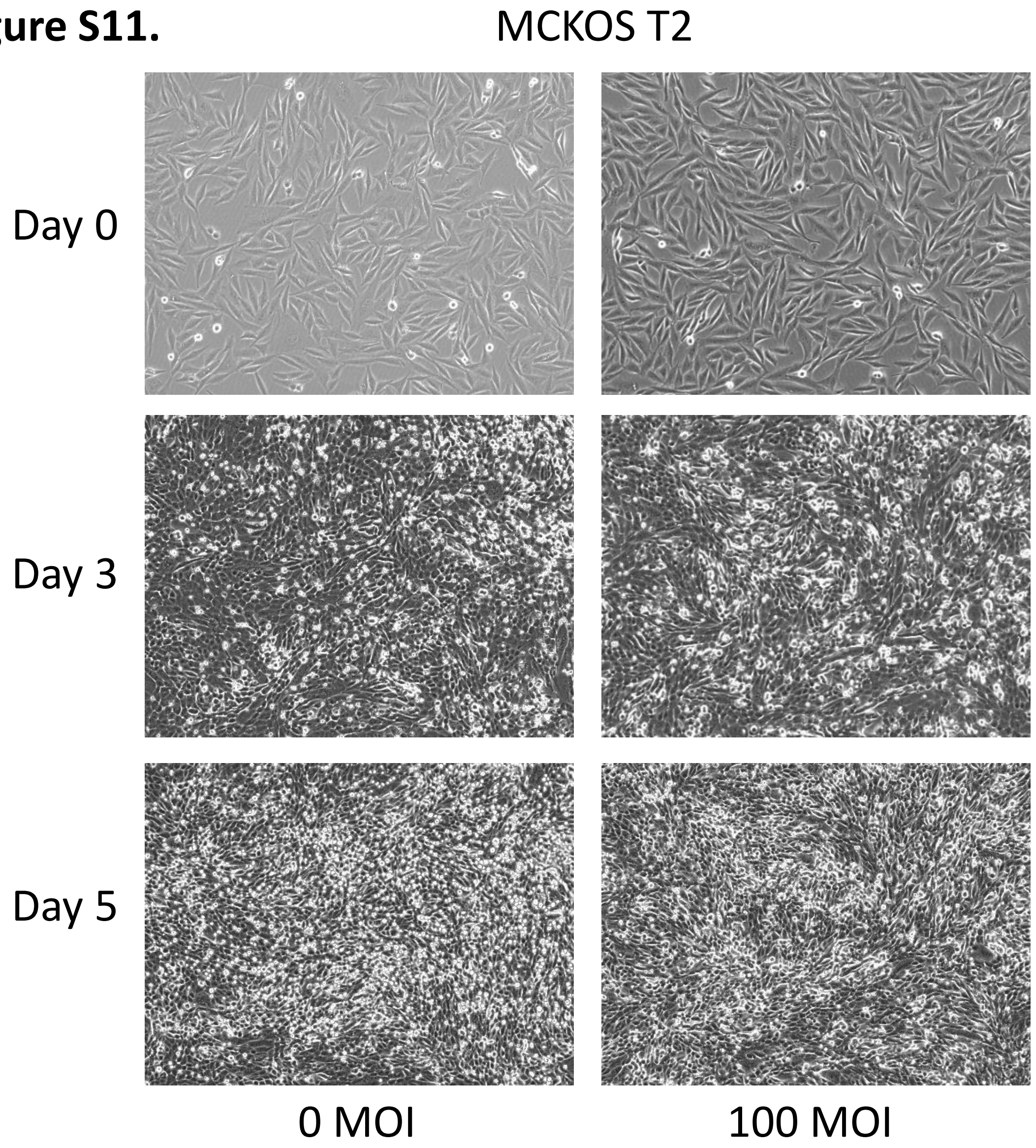

MCKOS cells were infected with CAV-AU-M3 at MOI 0 and 100, and 1000, and images were visualized on day 0, 3, and 5 post-infection by inverted microscopy (Keyence) at 10X magnification. This represents three independent experiments.

Figure S12.

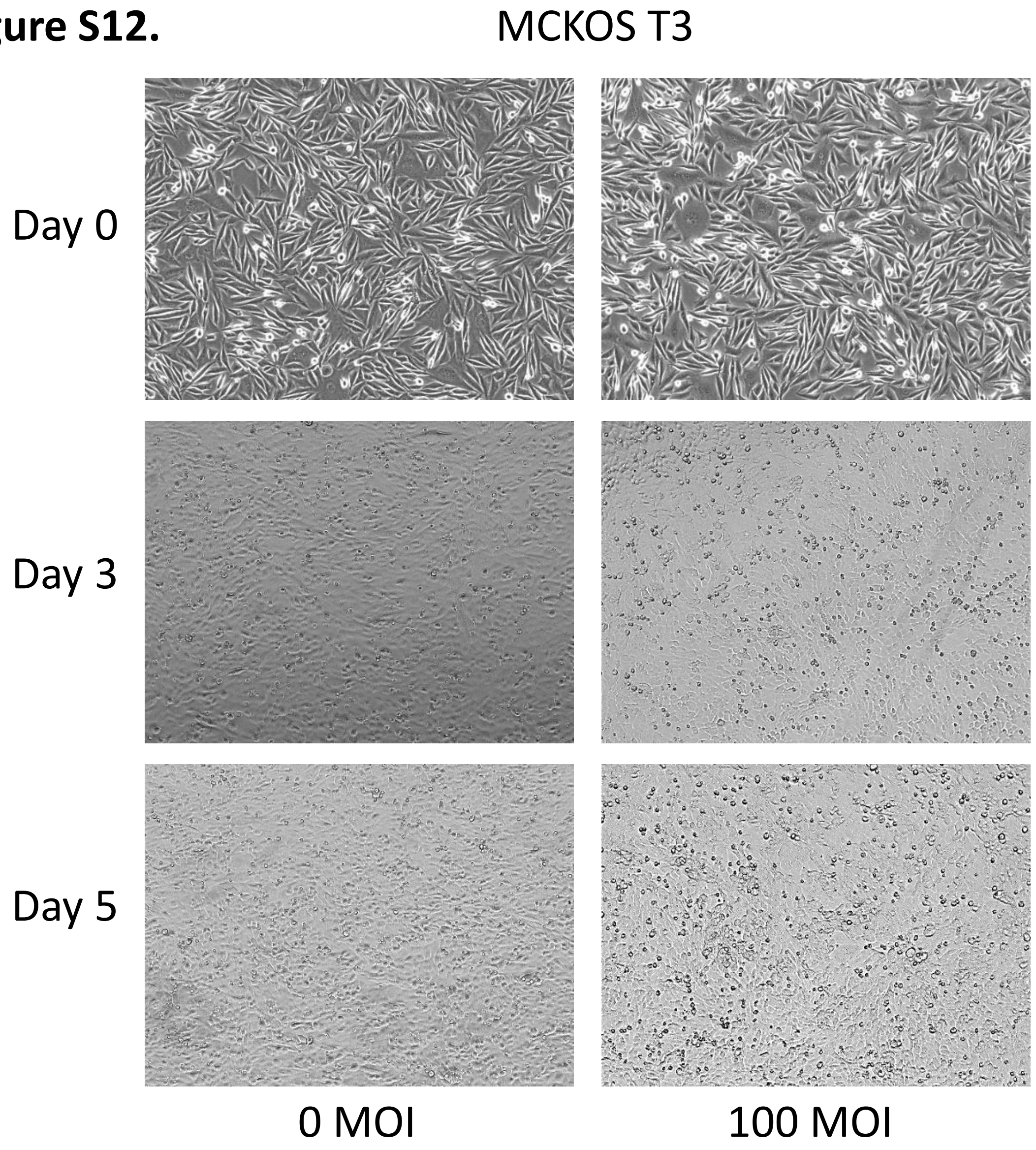

MCKOS cells were infected with CAV-AU-M3 at MOI 0 and 100, and 1000, and images were visualized on day 0, 3, and 5 post-infection by inverted microscopy (Keyence) at 10X magnification. This represents three independent experiments.

Figure S13.

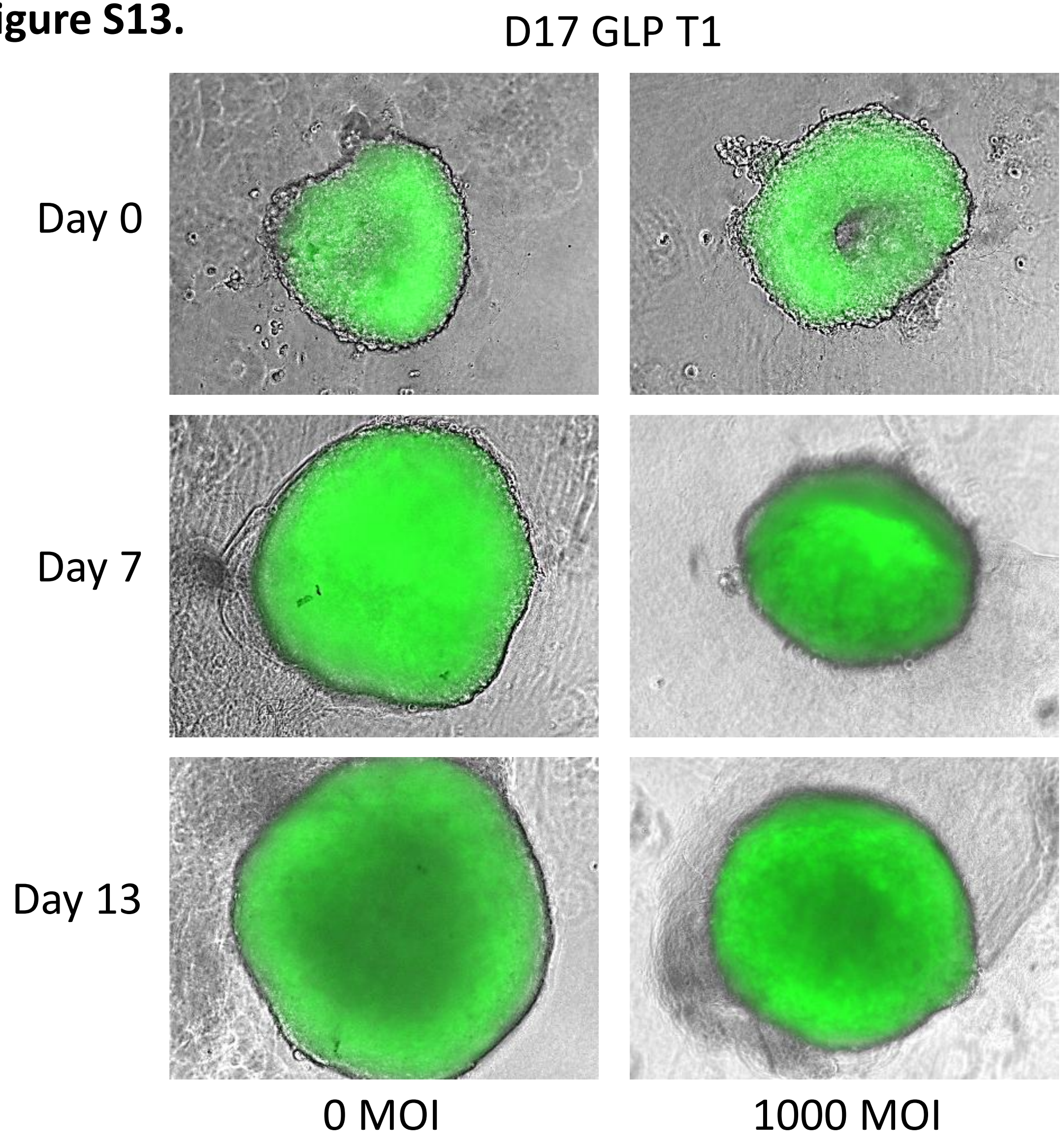

D17 GLP cell lines spheroids were infected with CAV-AU-M3 at MOI 0 and 1000, and the spheroid shrinkage and cytopathic effects were visualized at Day 0, 7, and 13 post-infection by fluorescence microscopy (Keyence) at 10X magnification. This is a representation of three independent experiments.

Figure S14.

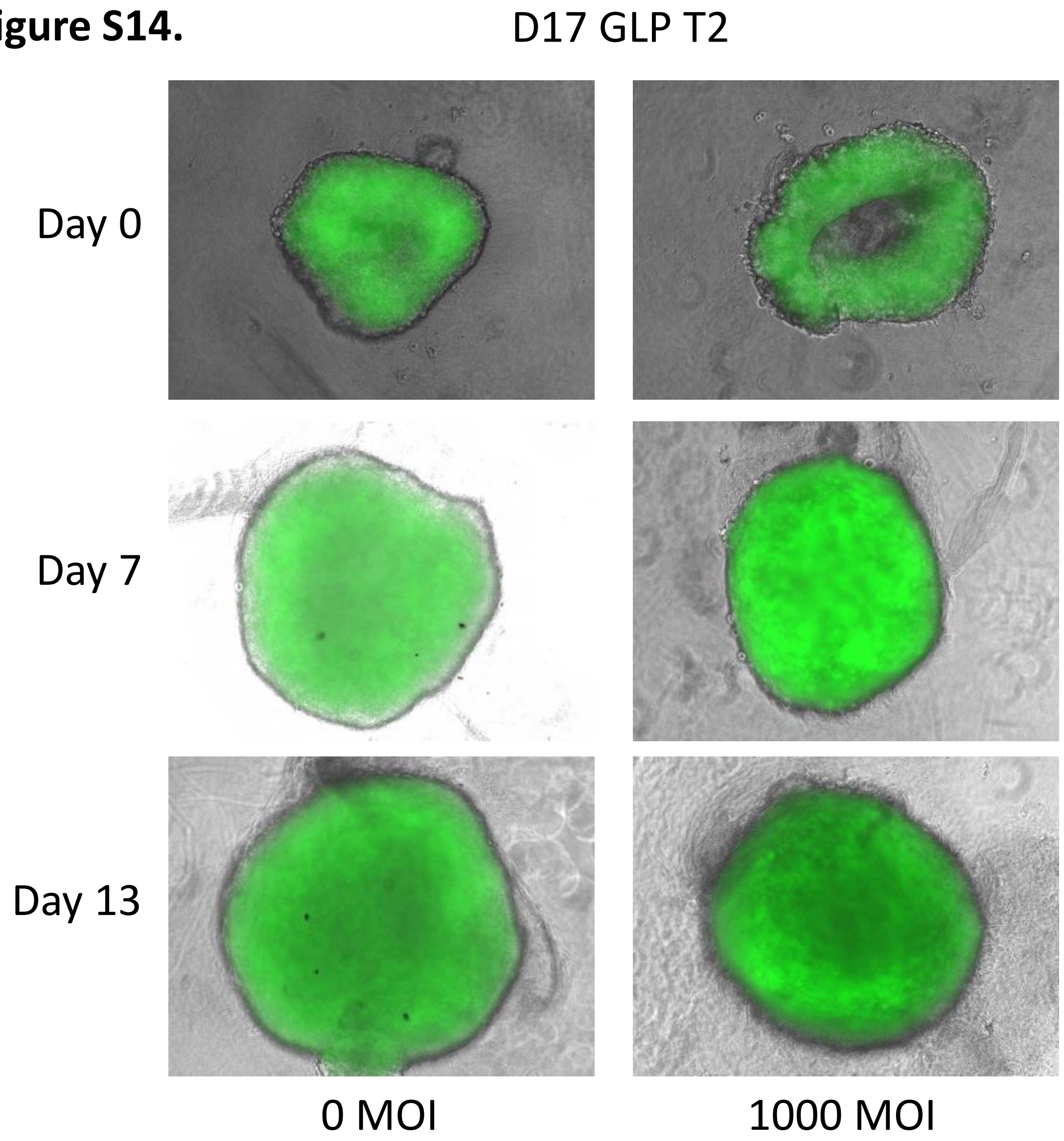

D17 GLP cell lines spheroids were infected with CAV-AU-M3 at MOI 0 and 1000, and the spheroid shrinkage and cytopathic effects were visualized at Day 0, 7, and 13 post-infection by fluorescence microscopy (Keyence) at 10X magnification. This is a representation of three independent experiments.

Figure S15.

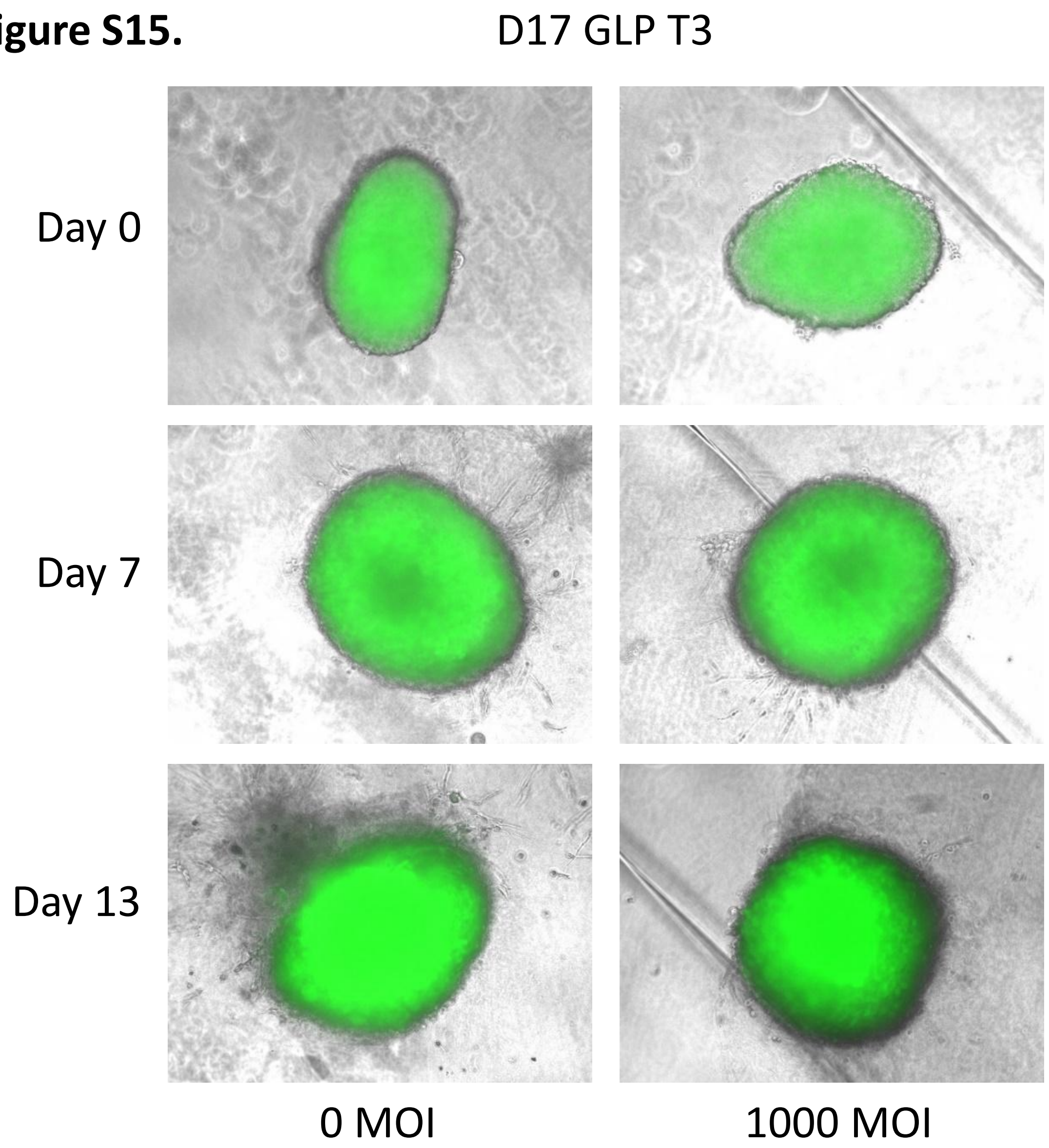

D17 GLP cell lines spheroids were infected with CAV-AU-M3 at MOI 0 and 1000, and the spheroid shrinkage and cytopathic effects were visualized at Day 0, 7, and 13 post-infection by fluorescence microscopy (Keyence) at 10X magnification. This is a representation of three independent experiments.

Figure S16.

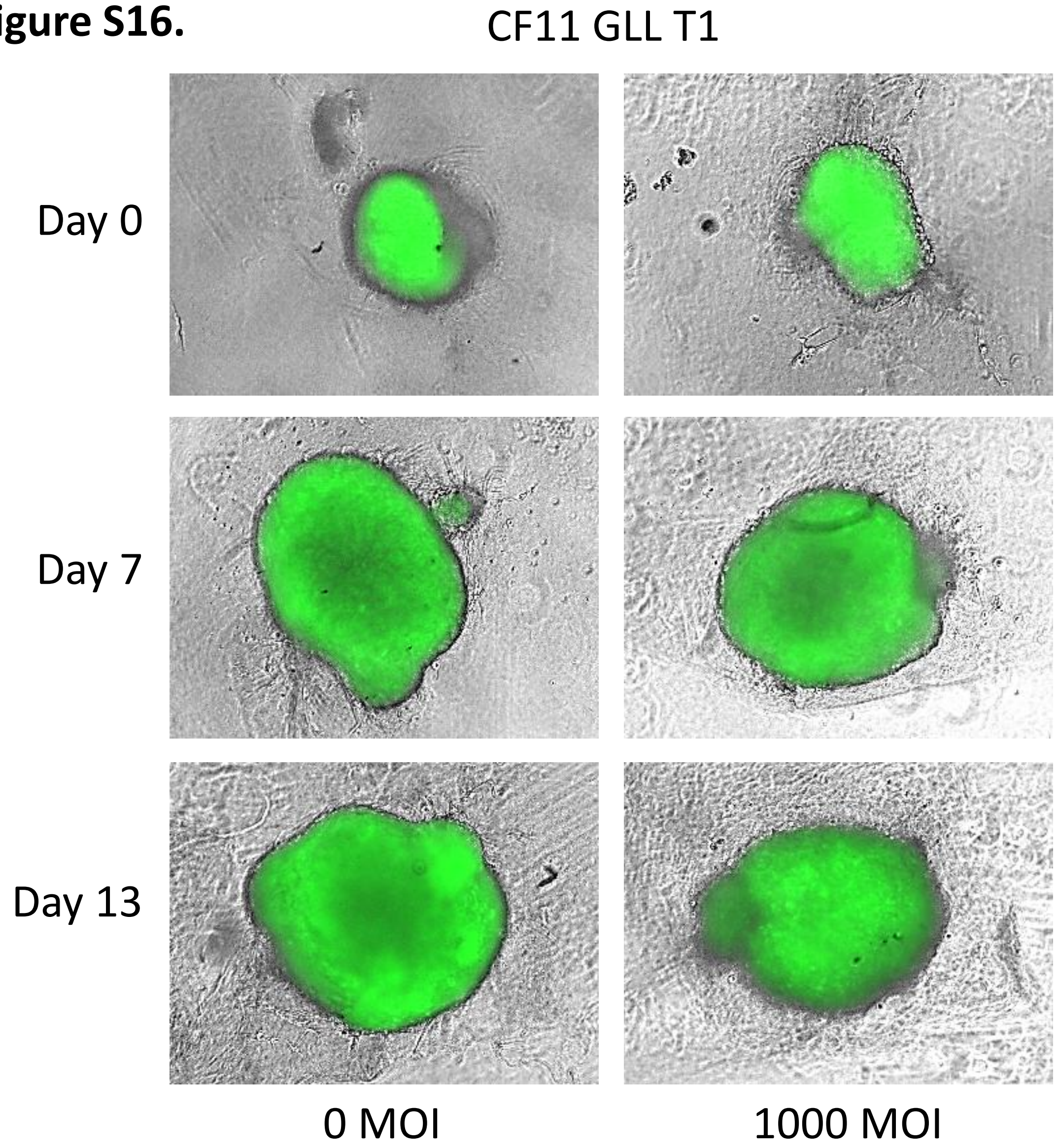

CF11 GLL cell lines spheroids were infected with CAV-AU-M3 at MOI 0 and 1000, and the spheroid shrinkage and cytopathic effects were visualized at Day 0, 7, and 13 post-infection by fluorescence microscopy (Keyence) at 10X magnification. This is a representation of three independent experiments.

Figure S17.

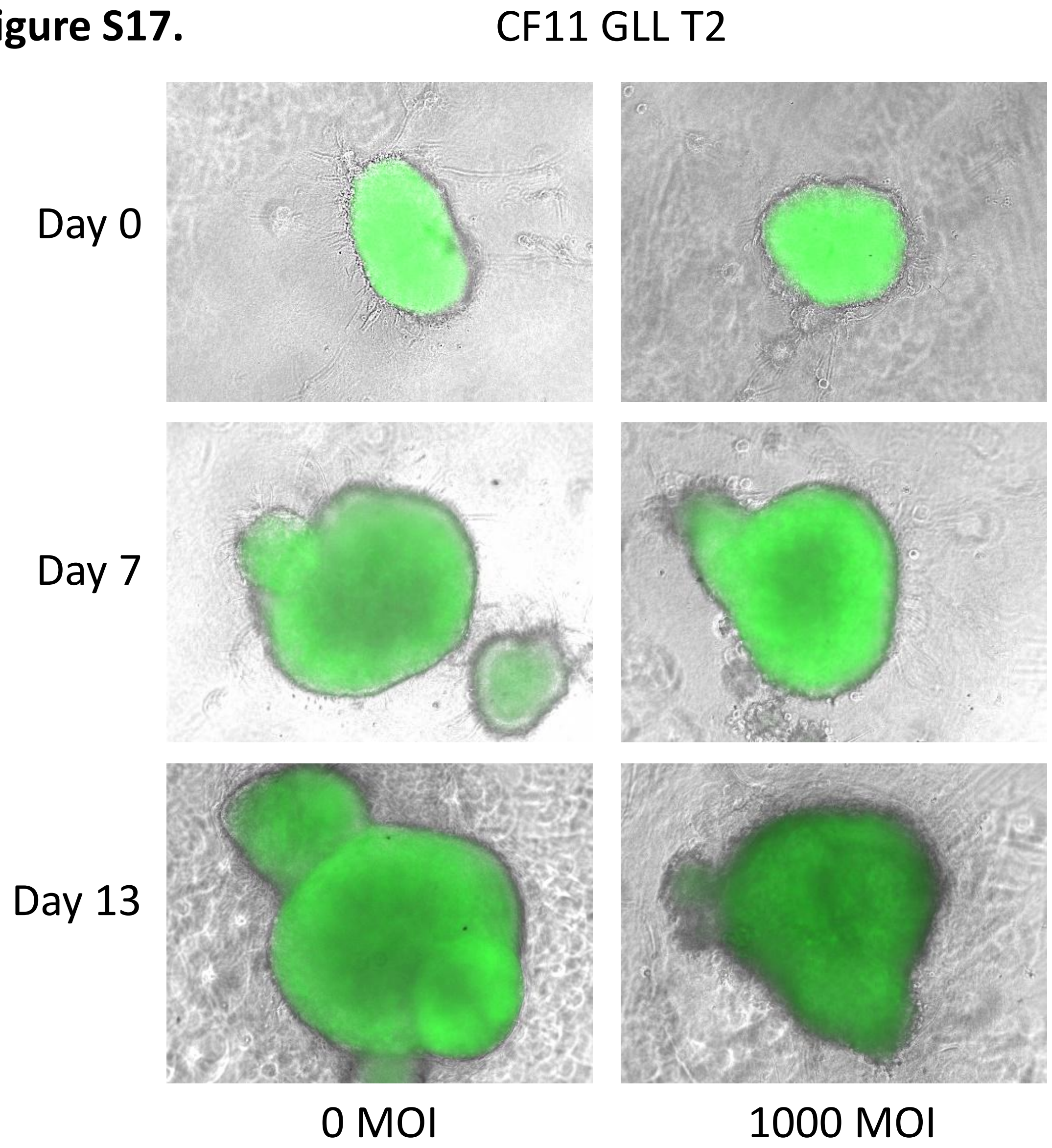

CF11 GLL cell lines spheroids were infected with CAV-AU-M3 at MOI 0 and 1000, and the spheroid shrinkage and cytopathic effects were visualized at Day 0, 7, and 13 post-infection by fluorescence microscopy (Keyence) at 10X magnification. This is a representation of three independent experiments.

Figure S18.

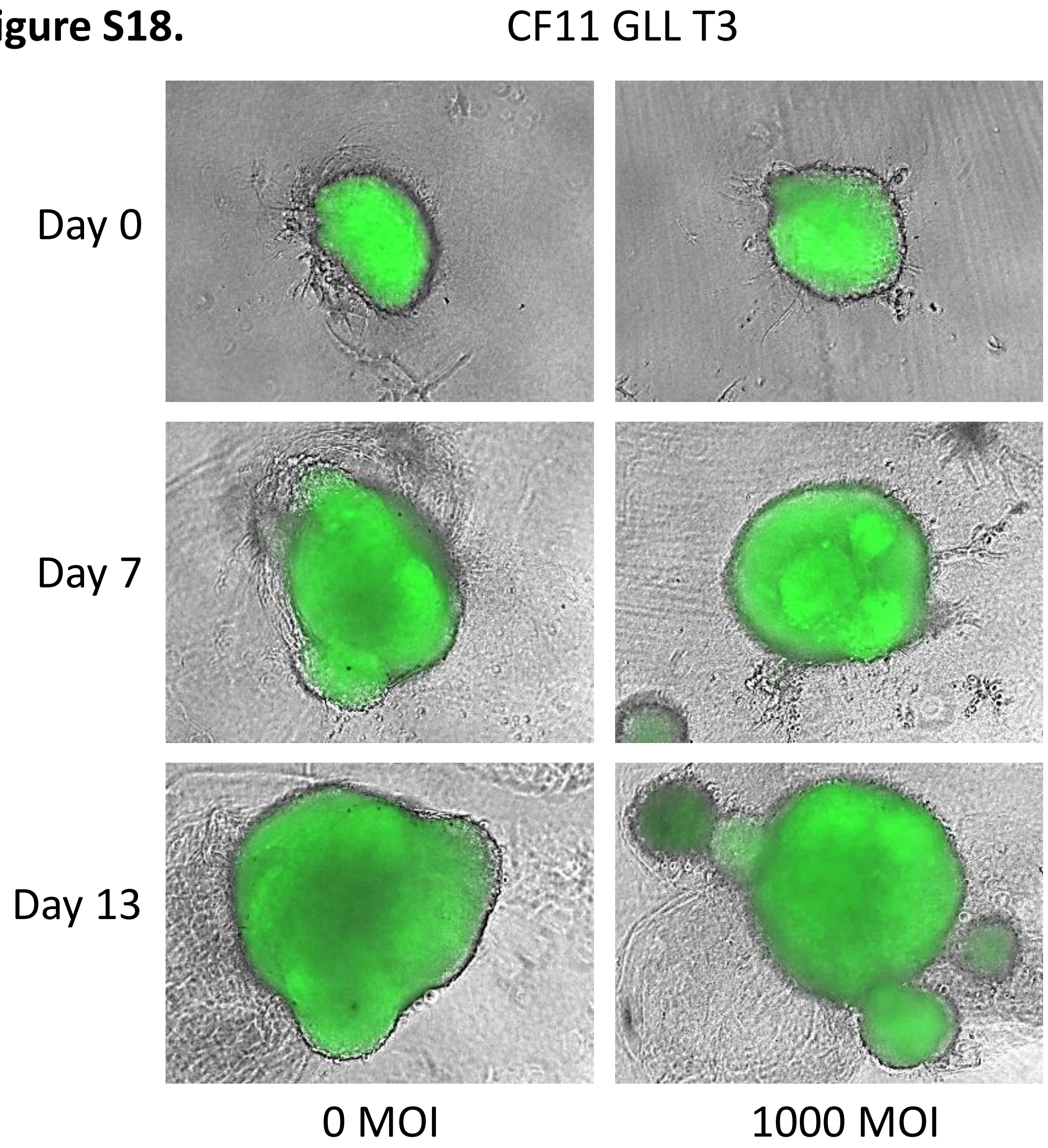

CF11 GLL cell lines spheroids were infected with CAV-AU-M3 at MOI 0 and 1000, and the spheroid shrinkage and cytopathic effects were visualized at Day 0, 7, and 13 post-infection by fluorescence microscopy (Keyence) at 10X magnification. This is a representation of three independent experiments.

Figure S19.

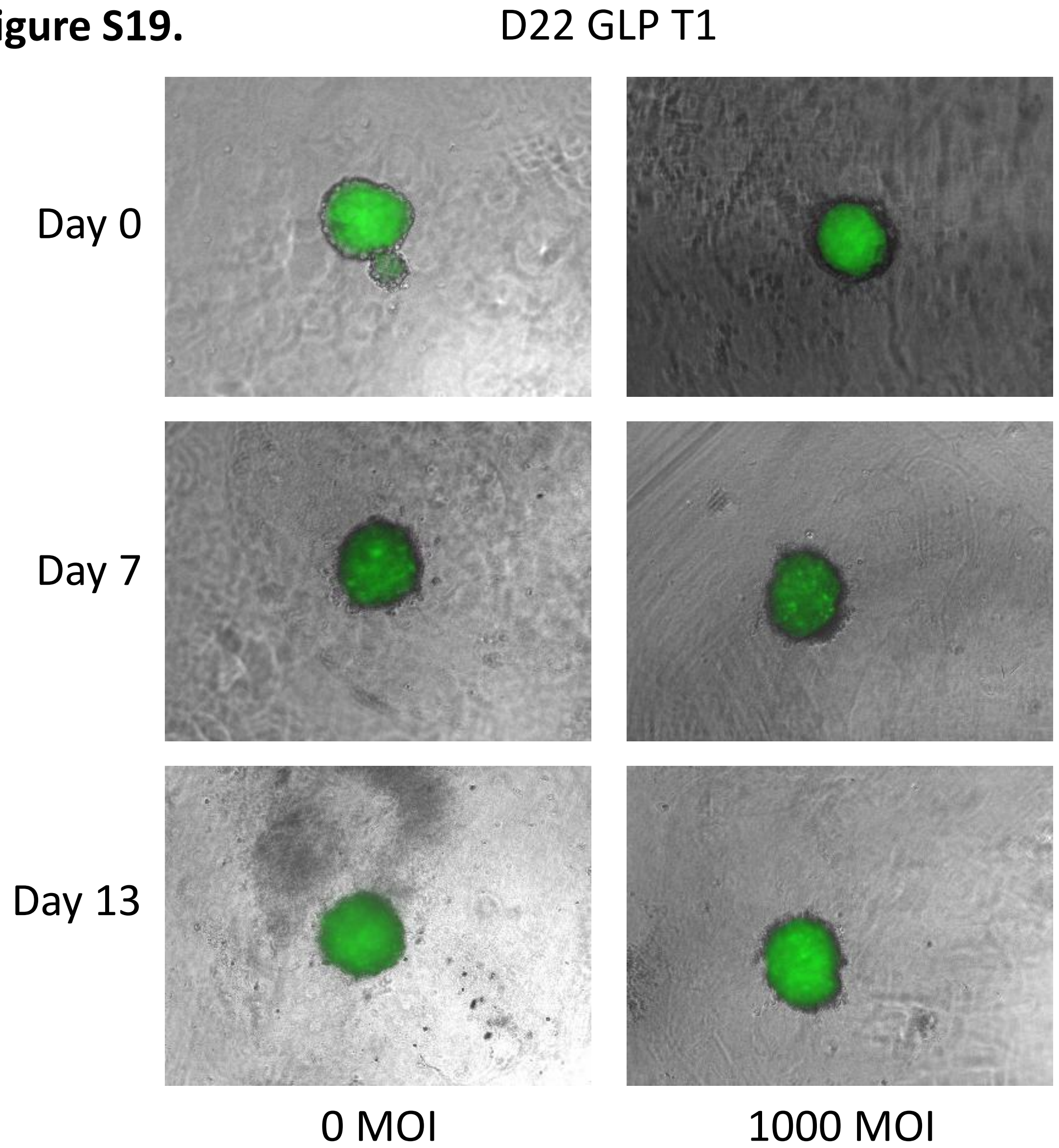

D22 GLP cell lines spheroids were infected with CAV-AU-M3 at MOI 0 and 1000, and the spheroid shrinkage and cytopathic effects were visualized at Day 0, 7, and 13 post-infection by fluorescence microscopy (Keyence) at 10X magnification. This is a representation of three independent experiments.

Figure S20.

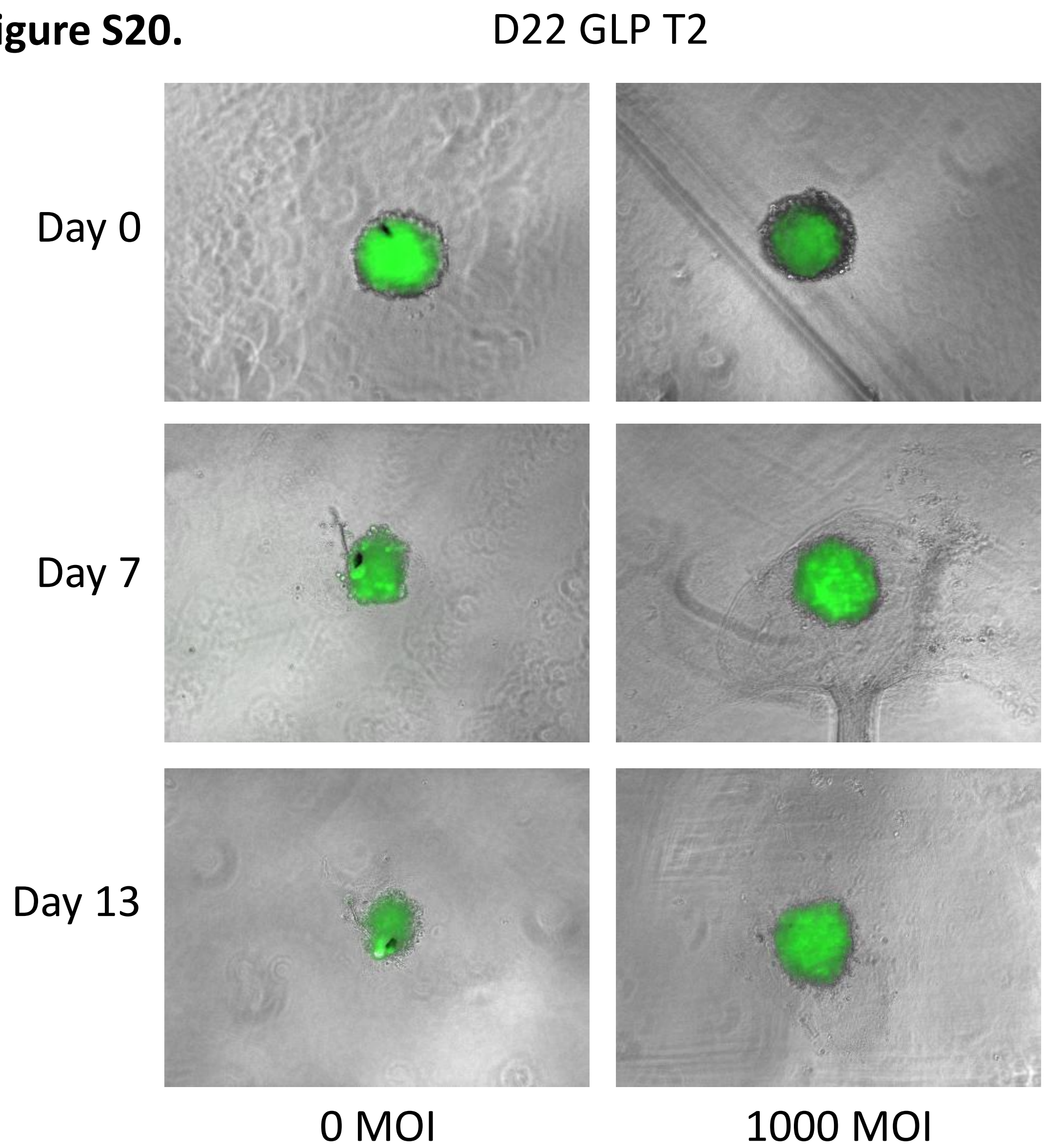

D22 GLP cell lines spheroids were infected with CAV-AU-M3 at MOI 0 and 1000, and the spheroid shrinkage and cytopathic effects were visualized at Day 0, 7, and 13 post-infection by fluorescence microscopy (Keyence) at 10X magnification. This is a representation of three independent experiments.

Figure S21. D22 GLP T3

Day 0

Day 7

Day 13

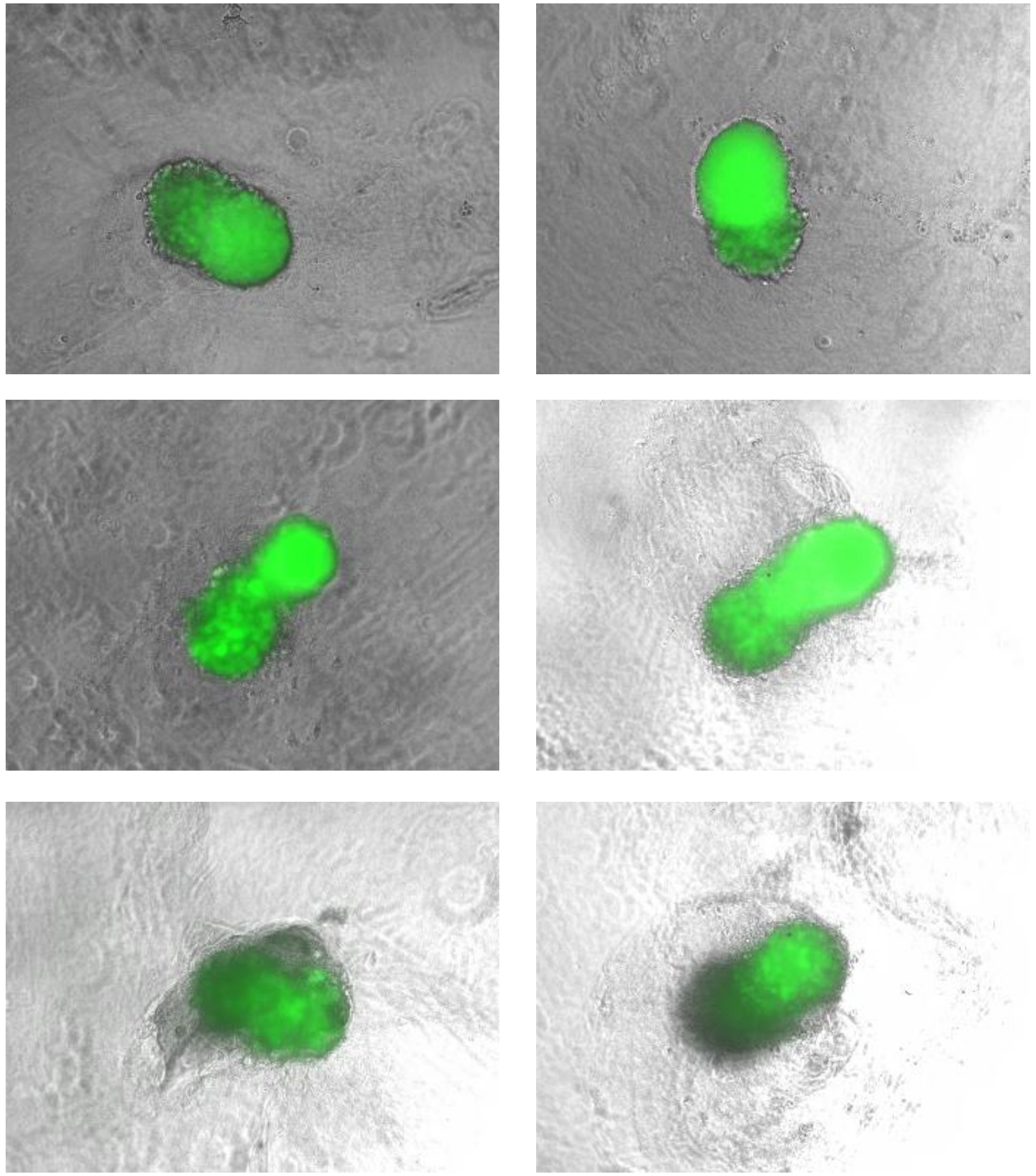

0 MOI 1000 MOI

D22 GLP cell lines spheroids were infected with CAV-AU-M3 at MOI 0 and 1000, and the spheroid shrinkage and cytopathic effects were visualized at Day 0, 7, and 13 post-infection by fluorescence microscopy (Keyence) at 10X magnification. This is a representation of three independent experiments.

Figure S22.

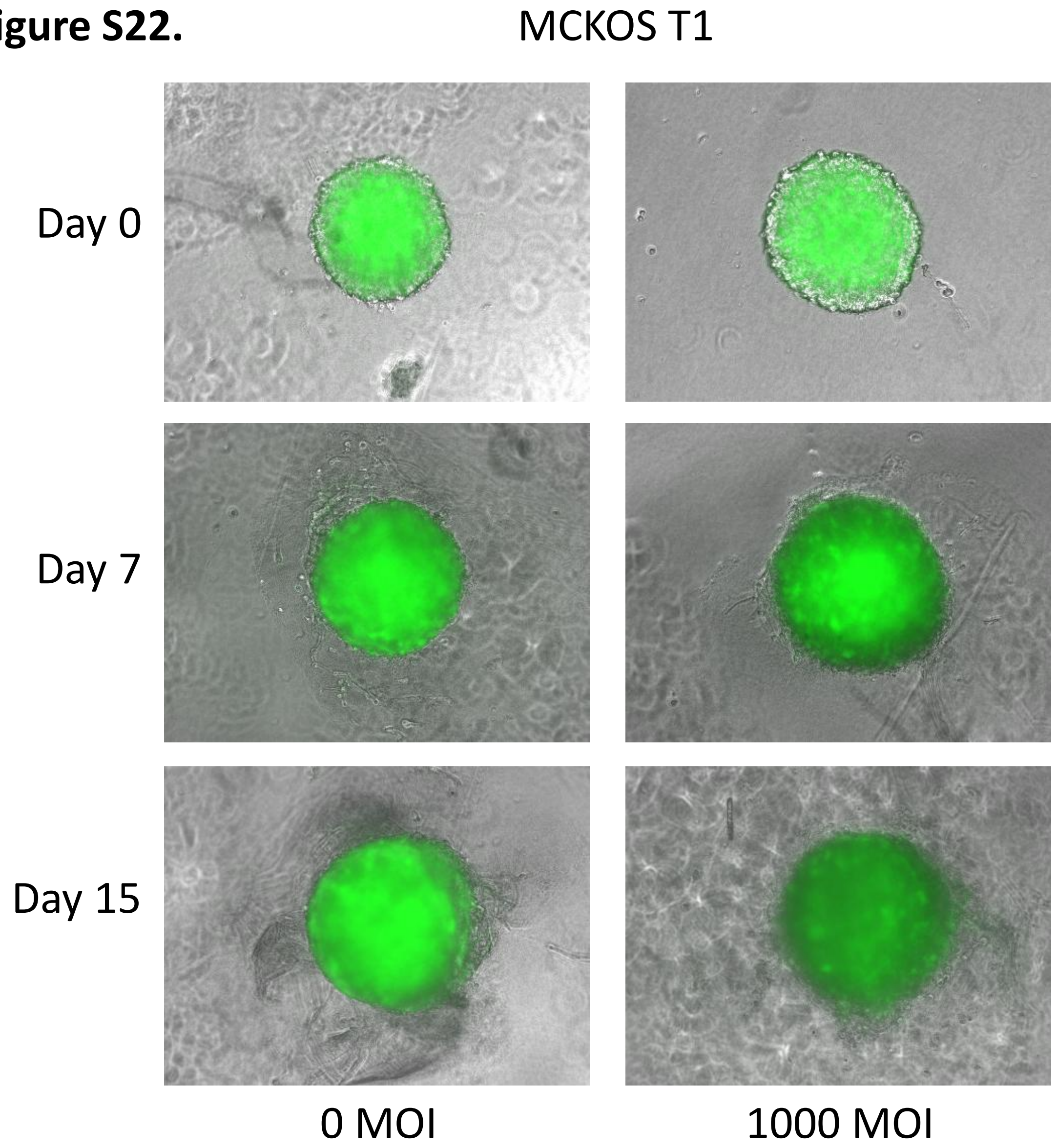

MCKOS cell lines spheroids were infected with CAV-AU-M3 at MOI 0 and 1000, and the spheroid shrinkage and cytopathic effects were visualized at Day 0, 7, and 13 post-infection by fluorescence microscopy (Keyence) at 10X magnification. This is a representation of three independent experiments.

Figure S23.

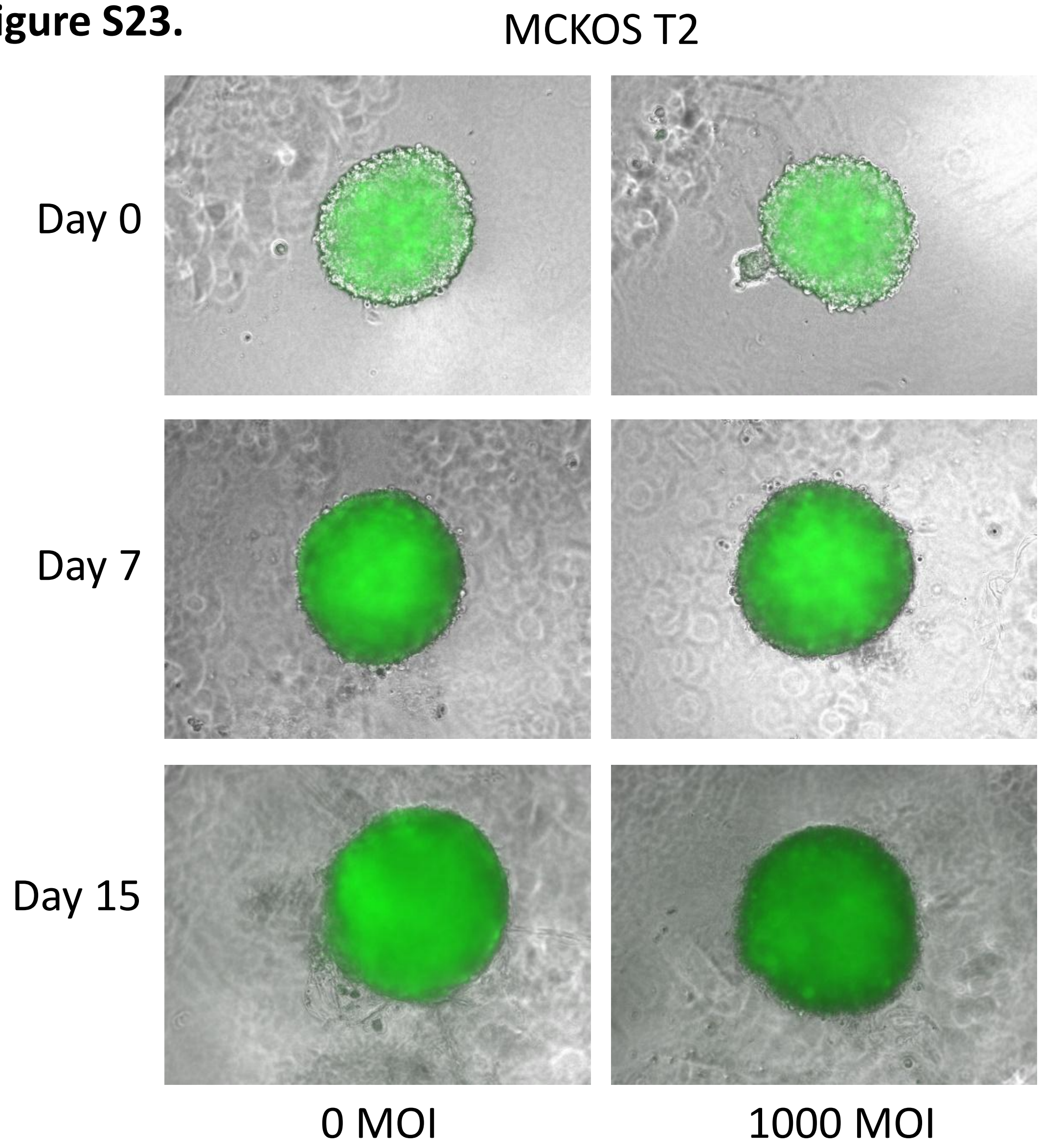

MCKOS cell lines spheroids were infected with CAV-AU-M3 at MOI 0 and 1000, and the spheroid shrinkage and cytopathic effects were visualized at Day 0, 7, and 13 post-infection by fluorescence microscopy (Keyence) at 10X magnification. This is a representation of three independent experiments.

Figure S23.

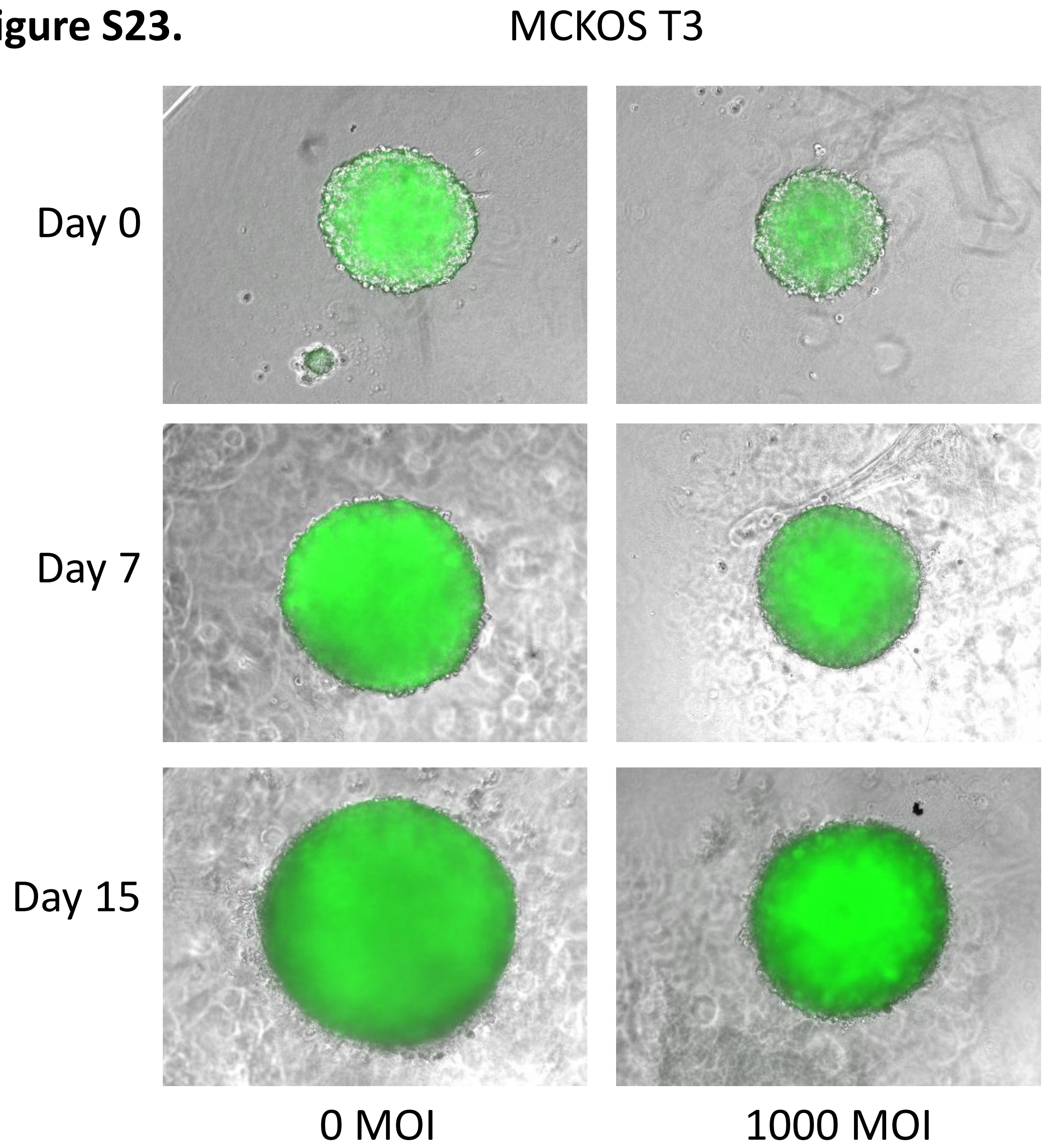

MCKOS cell lines spheroids were infected with CAV-AU-M3 at MOI 0 and 1000, and the spheroid shrinkage and cytopathic effects were visualized at Day 0, 7, and 13 post-infection by fluorescence microscopy (Keyence) at 10X magnification. This is a representation of three independent experiments.

Figure S24.

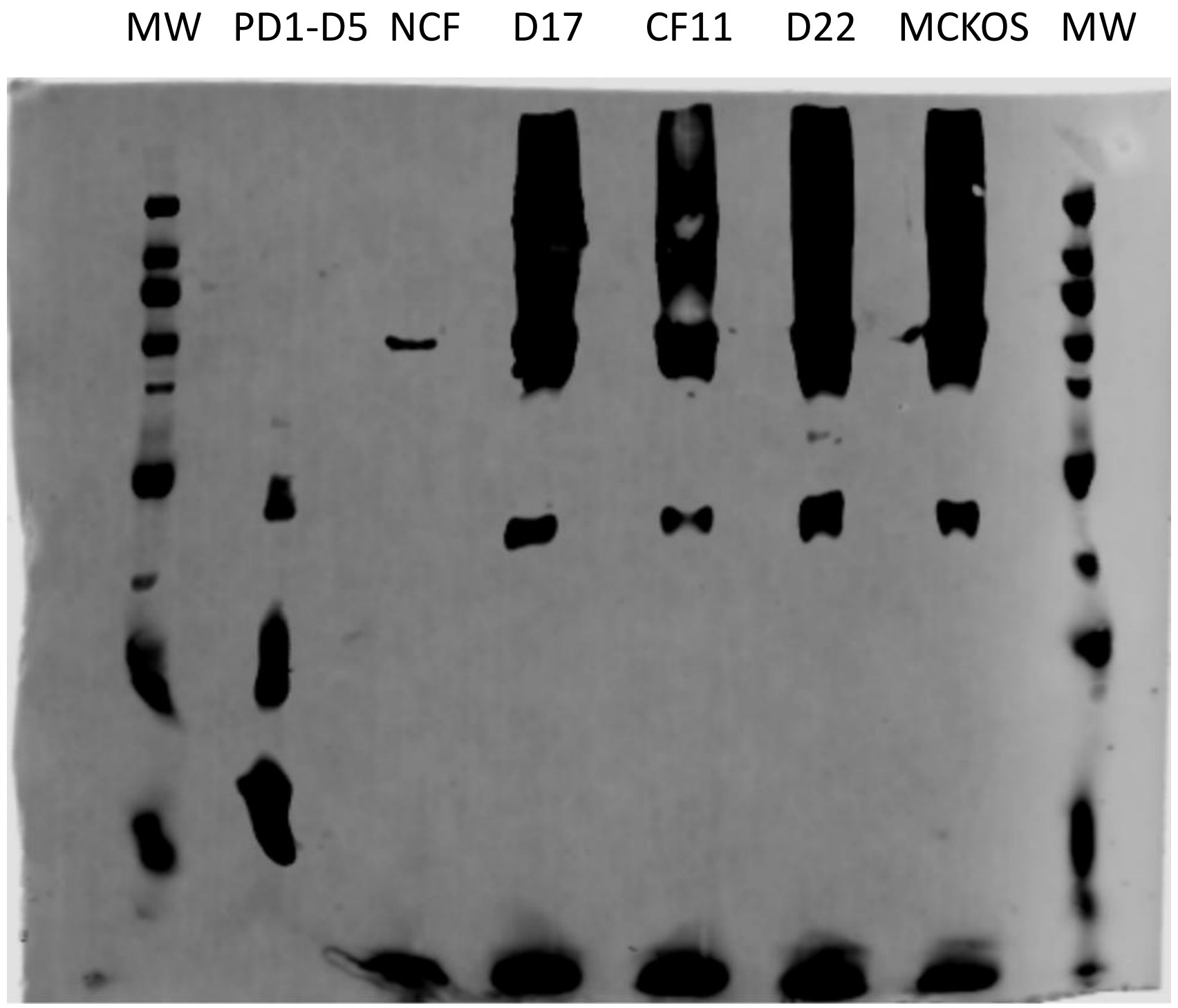

Western blot analysis: anti-PD1 HcAb was isolated from D17 GLP, CF11 GLL, D22 GLP, and MCKOS cell lines. Primary antibody: Mouse Anti-His, secondary antibody: anti mouse IgG

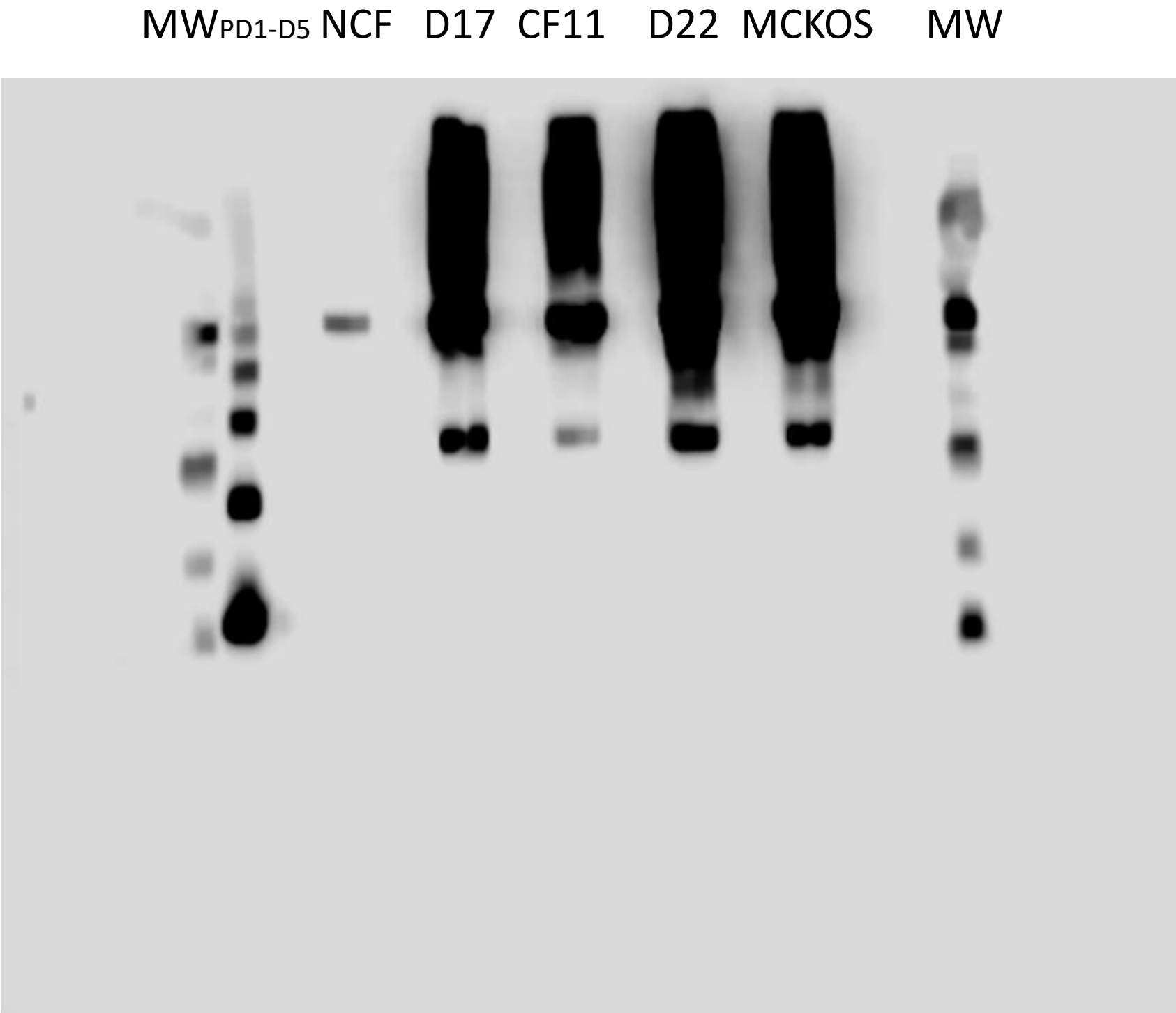

Western blot analysis: anti-PD1 HcAb was isolated from D17 GLP, CF11 GLL, D22 GLP, and MCKOS cell lines. Primary antibody:
Mouse anti-VHH IFlour 647
